# Structure of social networks of people living with HIV and AIDS*

**DOI:** 10.1590/1980-220X-REEUSP-2021-0525

**Published:** 2022-02-23

**Authors:** Séfora Luana Evangelista de Andrade, Maria Eliane Moreira Freire, Neusa Collet, Gisetti Corina Gomes Brandão, Maria Helena do Nascimento Souza, Jordana de Almeida Nogueira

**Affiliations:** 1 Universidade Federal da Paraíba, Programa de Pós-graduação em Enfermagem, João Pessoa, PB, Brazil.; 2 Universidade Federal da Paraíba, Programa de Pós-graduação em Enfermagem, Departamento de Enfermagem Clínica, João Pessoa, PB, Brazil.; 3 Universidade Federal da Paraíba, Programa de Pós-graduação em Enfermagem, Departamento de Enfermagem de Saúde Coletiva, João Pessoa, PB, Brazil.; 4 Universidade Federal da Campina Grande, Unidade Acadêmica de Enfermagem, Campina Grande, PB, Brazil.; 5 Universidade Federal do Rio de Janeiro, Escola de Enfermagem Anna Nery, Departamento de Enfermagem e Saúde Pública, Rio de Janeiro, RJ, Brazil.

**Keywords:** Acquired Immunodeficiency Syndrome, HIV, Social Support, Social Networking, Síndrome de Inmunodeficiencia Adquirida, VIH, Apoyo Social, Red Social, Síndrome de Imunodeficiência Adquirida, HIV, Apoio social, Rede social

## Abstract

**Objective:**

To analyze the structure of the social network of people living with HIV and AIDS.

**Method:**

Exploratory and descriptive research with a qualitative approach, developed through interviews with twenty-two people living with HIV and AIDS, from November to December 2019. For analysis, the theoretical-methodological framework of social network was used.

**Results:**

The primary networks were of medium size and low density, formed by family members, relatives, friends, neighbors, and colleagues. The secondary networks were characterized by public, private, third sector institutions, workplaces, and by the informal network, which provided support according to the need for care.

**Conclusion:**

The family was considered the center of the primary social network structure; however, weaknesses in these social relationships were evidenced. The family relational context of the person with HIV and AIDS was influenced by the secrecy of the diagnosis due to the fear of prejudice and discrimination for being HIV-positive. There was a predilection for the services of the secondary social network that took on the role of specific care for the disease.

## RESUMO

**Objetivo:**

Analisar a estrutura da rede social de pessoas vivendo com HIV e Aids.

**Método:**

Pesquisa exploratória e descritiva de abordagem qualitativa, desenvolvida por meio de entrevista com vinte e duas pessoas vivendo com HIV e Aids, de novembro a dezembro de 2019. Para análise, utilizou-se o referencial teórico-metodológico de rede social.

**Resultados:**

as redes primárias configuraram-se de tamanho médio e com baixa densidade, formadas por familiares, parentes, amigos, vizinhos e colegas. As redes secundárias foram caracterizadas por instituições públicas, privadas, de terceiro setor, locais de trabalho e pela rede informal, que forneceram suporte conforme a necessidade de cuidado.

**Conclusão:**

A família foi considerada o nó central da estrutura da rede social primária; no entanto, foram evidenciadas fragilidades nessas relações sociais. O contexto relacional familiar da pessoa com HIV e Aids sofreu influência do sigilo do diagnóstico decorrente do medo de sofrer preconceito e discriminação por ser soropositiva. Houve predileção pelos serviços da rede social secundária que assumiram a função de cuidado específico para a doença.

## RESUMEN

**Objetivo:**

Analizar la estructura de la red social de personas viviendo con VIH y SIDA.

**Método:**

Investigación exploratoria y descriptiva de abordaje cualitativo, desarrollada por medio de entrevista con veintidós personas viviendo con VIH y SIDA, de noviembre a diciembre de 2019. Para tal análisis, se utilizó el referencial teórico-metodológico de la red social.

**Resultados:**

Las redes primarias se configuraron de tamaño medio y con baja densidad, formadas por familiares, parientes, amigos, vecinos y colegas. Las redes secundarias se caracterizaron por instituciones públicas, privadas, del tercer sector, locales de trabajo y por la red informal, que aportaron soporte de acuerdo con la necesidad de cuidado.

**Conclusión:**

La familia fue considerada el eje de la estructura de la red social primaria, sin embargo fueron evidenciadas debilidades en esas relaciones sociales. El contexto relacional familiar de la persona con VIH y SIDA tuvo influencia del sigilo del diagnóstico decurrente del miedo de sufrir perjuicio y discriminación por su condición seropositiva. Hubo preferencia por los servicios de la red social secundaria que asumieron la función de cuidado específico para la enfermedad.

## INTRODUCTION

In general, diseases lead to changes in the way people deal with their routine, social, work and family networks, as well as necessary changes in self-care, since they generate doubts and feelings of insecurity, fear and anxiety^(1)^. With the diagnosis of HIV would be no different. For many people, it is a traumatic experience that can trigger psychic suffering, associated with several experiences, including fear of death and stigma^(2)^.

Furthermore, the social and emotional impact of the diagnosis of HIV is highlighted, as it carries, in addition to situations of coping with discrimination due to the multidimensional nature of the disease-related stigma^(3)^, challenges caused by changes in social relationships in the context of work, family and community life, factors that end up compromising treatment adherence^(4)^.

The production of care in the HIV and AIDS care setting shall transpose the technical and/or standardized perceptions^(5)^. The challenge posed requires the use of devices that allow for better adaptation and coping with the problem, focusing on these individuals’ health condition and social relationships.

The social network approach can bring significant contributions to studies with people living with HIV and AIDS, as it includes new facets not seen through traditional analyses^(6)^. This type of study allows understanding how these networks influence the actions taken by individuals in view of their health needs^(7)^.

Social networks can be understood as the set of interpersonal relationships of an individual that define their characteristics (habits, traditions, beliefs, values), responsible for their social identity and for shaping their social relationships^(8)^. Social networks can be primary and secondary, differing in the types of interactions between individuals^(8)^.

Primary networks are made up of family, kinship, friendship and neighborhood ties, with bonds based on reciprocity and trust^(8)^. Secondary networks, on their turn, are divided into formal and informal. The formal one comprises the set of state institutions forming the population’s social welfare system, including the third sector (civil society organizations, which operate on a non-profit basis), the market (economic activities) and mixed ones (provide services, guaranteeing rights, upon payment). Finally, the informal secondary networks comprise an unfolding of the primary networks, constituted by mutual aid groups, with non-formalized ties and functions based on verbal agreements^(8)^.

The structure of the social network comprises the set of perceptible ties established between people and networks, and, when these ties are activated, they generate connections that shape the networks. An adequate social network in its extension and quality of relationships plays a role of support or containment in the face of various personal and social demands^(8)^. Individuals who have a wider social support network can develop more resilience, using more psychological and protection resources against the adversities that appear with HIV disease^(9)^.

Studies have revealed spouses or partners, family members, in or out of the family environment, and friends as the main sources of social support for people living with HIV and AIDS^(10–12)^. However, despite the scarcer offer of support, colleagues, neighbors, bosses at work, and the health team are also referred to as sources of support^(10–11,13)^.

Social support provided, either emotional and/or ­instrumental, impacts on acceptance of the HIV diagnosis; favors adherence and continuity of treatment; improves mental health; mitigates the effect of stigma in these people’s daily lives, contributing to coping with the disease with a better quality of life^(10–11, 14–15)^.

This context implies the need for interventions that are less technocratic and more sensitive to the subjectivities of people living with HIV and AIDS. It is essential to understand how socially deep-seated inequalities and stigmas produce and reproduce excluding behaviors with negative reflexes on these people’s physical and psychological well-being. Therefore, this study is justified by the need to know the constitution of the social networks of this population, through its description and structural analysis, since these relationships impact these individuals’ daily lives, their social and health demands and, therefore, shall be valued during the planning and implementation of care actions. Thus, the objective was to analyze the structure of the social networks of people living with HIV and AIDS.

## METHOD

### Design of Study

This is an exploratory, descriptive study, with a qualitative approach, directed by the guidelines of the *Consolidated criteria for reporting qualitative research* (COREQ) and based on the theoretical-methodological framework of social network by Lia Sanicola^(8)^.

### Local

The research was carried out at the outpatient clinic of the state hospital that is reference in assistance to people with infectious diseases, located in João Pessoa-Paraíba.

### Selection Criteria

People living with HIV and AIDS, attended at the state reference outpatient clinic during the period for collecting ­information, and living in the city of João Pessoa-PB, ­participated in the study. Those under 18 years old were excluded.

### Data Collection

The collection of information took place through a semi-structured interview, carried out at the outpatient clinic in a private environment, from November to December 2019. The participants were interviewed by an investigator, a doctoral student in nursing, with experience in the data collection ­technique. The script contained information on the characterization of the participants and the following guiding questions: What is the structure of the social network of people living with HIV and AIDS? How does the relationship between people living with HIV and AIDS and their social network take place?

**Figure 1:**
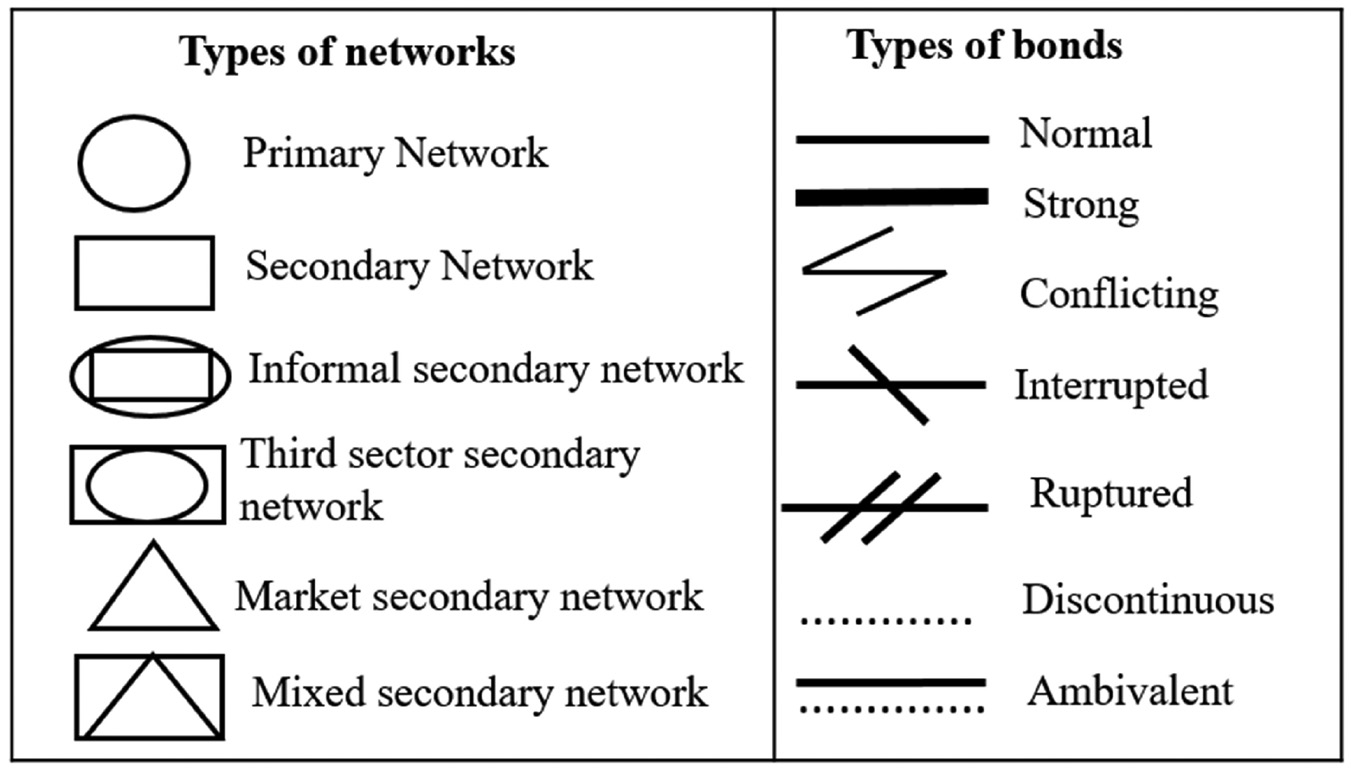
Geometric representation of social network types.

Source: Sanicola, 1995 (adapted).

**Figure 2:**
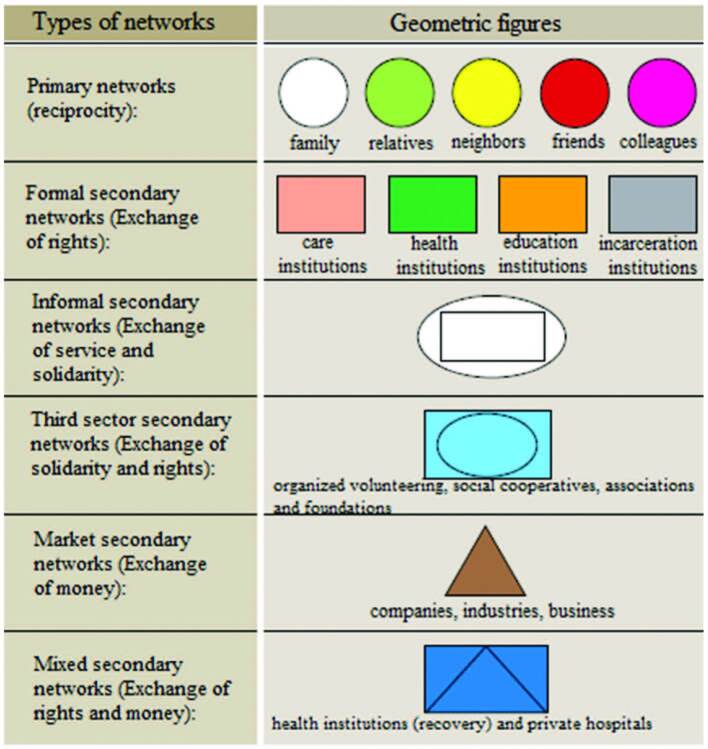
Representation of social network types.

Source: Soares (2002).

The networks map design took place during the interviews, being made with the participant. The graphic style adopted in the research is called *Rousseau’s map* and was built following the charts with the graphical representations of the types of network and links (Figure 1). The design was constructed on an A4 sheet of paper and colored with colored pencils, according to the model adapted by Soares^(16)^, shown in Figure 2.

The interviews were recorded and then transcribed by the main investigator. The participants’ anonymity was ensured, and they were identified by a chosen pseudonym; the other members mentioned in the network map design were identified according to the degree of kinship or their social relationship with the interviewee. The end of the collection met the sufficiency criterion, which comprises the moment in which it is possible to draw a comprehensive picture that responds to the objective and questions of the study^(17)^.

The field notes allowed real time recording of expressions, attitudes, facts perceived in the research field, and the medical records were consulted to extract personal data (age, date of diagnosis) and origin (address).

All maps of the participants’ social networks were ­designed by the main investigator using *Draw.io*, an online diagram editor. Then, a map was built with the consolidation of the ­individual networks.

### Data Analysis and Treatment

The structural analysis of the social networks described, aided by the maps, was carried out through the elaboration of the network map, aiming at getting to know the dimension and the way in which the ties are established between the different types of network present^(8)^.

To analyze the social network, it was necessary to know indicators of its structure in terms of amplitude – with regard to the number of people present, allowing us to consider that a network is small (up to nine), medium (ten to thirty), or large (more than thirty members); of density – which refers to the types of ties established, considered high (all members of the network know each other), medium (bonds only between some of the members mentioned), and low (few or none of the members maintain bonds or know each other)^(8,16)^. In the maps analysis, the source of the support provided by the members of the networks was evidenced.

### Ethical Aspects

The study was approved by the Research Ethics Committee under opinion 3.667.428/2019. The investigators were compliant with the ethical aspects of research involving human beings according to guidelines of Resolution 466/12 of the National Health Council^(18)^. The participants selected were initially invited and, after acceptance, gave their consent by signing the Free and Informed Consent Form (FICF).

## RESULTS

Twenty-two people participated in the study, of which 15 had HIV and seven had AIDS, with 11 men and 11 women. All were under outpatient care at the specialized service in the state of Paraíba, with time since HIV diagnosis ranging from one month to 20 years. Regarding age, the men participating in the study were between 23 and 58 years old, and the women between 19 and 63 years old.

At the time of collection, most were single, self-declaring themselves mixed race and of Catholic religion. Regarding the profession, most women were unemployed, retired, or were housewives, while most male participants were included in the job market, in various professions, such as lawyer, businessman, service supervisor, attendant, among others.

In the analysis of individual primary networks, of the 22 social network maps, 13 were configured with medium amplitude (10 to 30 members), as they ranged from 10 to 23 members. It should be noted that no primary social network map was configured as large (more than 30 members).

It is worth noting that out of nine people with a longer HIV diagnosis, from five to 20 years, five had primary networks characterized with a small amplitude, with three to nine ­members, suggesting that having a longer time of experience of the disease does not contribute directly in the construction and presentation of broader primary networks.

Regarding the density indicator of primary networks, the most frequent was the low one, identified in 12 networks. Only three maps were characterized with high density, evidencing fragility in the participants’ social relationships.

**Figure 3:**
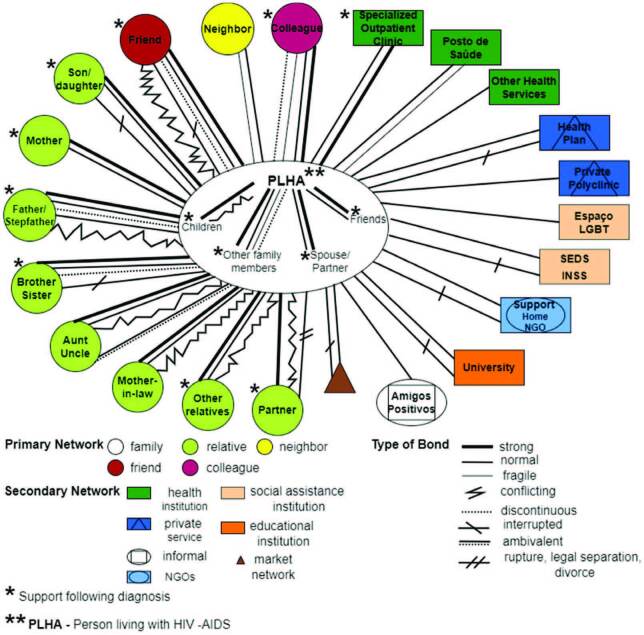
Consolidated map of the social networks of 22 people living with HIV and AIDS.

Knowing that there was a diversity of actors/sectors and bonds in the representation of the social networks of people living with HIV and AIDS, Figure 3 presents the ­consolidated map of the social networks of the 22 ­participants. For this purpose, all the social relationships built on the ­participants’ individual maps were considered, including all the members mentioned in the primary networks, as well as all the institutions that made up the secondary network.

The bonds established included different types: *normal, strong, fragile, conflicting, discontinuous, interrupted, ­ruptured and ambivalent*, being arranged according to the member referred to; therefore, in the figure, there is more than one line. Participants traced the nature of these bonds considering coexistence, ­affective and/or physical proximity, help with needs, as well as difficulties in family and social relationships.

The primary network of people living with HIV and AIDS is made up of family members, relatives, friends, colleagues and neighbors. In general, they live with children, a partner or ­husband, other family members (mother, father, sibling, nephew, grandson, brother-in-law, and cousin) and friends. Kinship ties are built with: mother, son/daughter, father/stepfather, partner, brother/sister, aunt/uncle, mother-in-law and other relatives (sister-in-law, son-in-law, daughter-in-law, husband’s family, cousin, nephew (niece), and grandson (daughter)).

The bonds of *strong* nature were motivated by situations of greater coexistence and/or help with needs, as well as greater physical and affective proximity. In the primary network, the construction of bonds of a *strong* nature with family members (mother, father, spouse, partner, children, nephew, grandchildren, siblings, son-in-law, brother-in-law) and friends with whom they live, as well as relatives (mother-in-law, aunt/uncle, brother/sister, cousin, and nephew (niece)), friends and co-workers.

In the primary network, the *normality* bond predominated with family members (partner, mother, father, child, brother/sister, niece) and friends they live with, aunt/uncle, mother-in-law, brother/sister, mother-in-law, other relatives (father, cousin, husband’s family, sister-in-law, nephew), colleague, ­neighbor, and friend.

The *fragile* bond was mentioned with the father, brother/sister, other relatives (aunt, daughter-in-law), neighbors, and colleagues, while the *conflicting* bond was referred with ­partner, son, aunt, stepfather and friends. The *discontinuity* bond was mentioned in the relationships with father, brother/sister, ­brother-in-law, ex-partner, friends, and colleagues. *Interrupted* bond appears in relationships with children, brother, uncle, and friend, and the *ruptured* bond with the husband.

Thus, the primary social network of people living with HIV and AIDS consists mainly of family members, relatives, and friends. However, the presence of the neighbor was evidenced in seven individual maps, although they were not mentioned as a source of support. Considering proximity, colleagues also made up this network, especially those from work, present in 16 individual networks.

It should be noted that the disclosure of the diagnosis was made only to some of the closest members of the networks, such as mother, father, children, sisters, boyfriend/partner, and close friends, most of them with *strong* bonds. However, two ­participants maintain total confidentiality and none of the ­members of their primary network is aware of the diagnosis of the disease.

Among the secondary network institutions, characterized by non-chosen relationships, there are the formal and informal ones, because they are institutional. The participants’ formal secondary network consists of health institutions (specialized outpatient clinic of the State Reference Hospital, Health Center and other services); education institutions (Universities); social assistance institutions (*Espaço LGBT*, National Institute of Social Security – *INSS*, Department of Social Development – *SEDS*); the third sector network (Support Houses and Non-Governmental Organization – NGO); mixed networks (Private Polyclinics and Health Plan services); and the market (jobs).

In addition to formal networks, one participant reported having had support through information about the disease in an online social network group, the *Amigos Positivos* (“Positive Friends”), characterized as an institutional bond and an informal network. In its individual primary network, a *discontinuous* bond with a colleague from this group was described.

Among the institutions of the formal secondary health network, the outpatient clinic of the reference Hospital, a state, secondary, and specialized service for care of people with HIV and AIDS and other communicable diseases, was mentioned in all 22 individual networks, characterized predominantly by bonds of *normality*, with medical professionals from different specialties and nursing team, but pharmacy, laboratory, and reception professionals were also mentioned. The *strong* bond with this institution was mentioned in two networks, specifically with infectious disease professionals. It should also be noted that this service was mentioned in 10 networks as the place for HIV diagnosis.

The primary care service, known as the *posto de saúde* (health center), also constitutes the secondary network of people living with HIV and AIDS, being mentioned in six individual networks. The bond was characterized as institutional, with no reference to specific professionals and, in some individual networks, the bonds were represented as *normal* and *fragile*. However, it should be noted that, in these networks, the health centers services were used for other needs, not related to HIV disease, such as for exams, consultations for children, dental consultations, among others.

Other health institutions that make up the secondary network of people living with HIV and AIDS consist of public services sought in the search for diagnosis and also for other needs not related to HIV and AIDS, such as treatment of rectal cancer, monitoring of osteoporosis, and other various medical consultations, including monitoring for Tuberculosis and syphilis.

Social institutions (*INSS* and *SEDS*) that are also part of the formal secondary network were included because they represent the main, and often the only, source of income for people living with HIV and AIDS, such as retirement, sickness aid, and family allowance (*Bolsa Família*), but with no bond with professionals, being configured as institutional bonds. For the *INSS*, there was an *interrupted* bond, due to the interruption of disability (illness) retirement.

Another social institution, *Espaço LGBT*, was included because it is the place where psychological care is provided. This bond was built due to the lack of timely vacancy for this type of service in the specialized outpatient clinic.

The mixed secondary network consists of services from the health plan and private institutions, which were included to make up the HIV diagnosis network in 4 individual networks, as well as for having the services used for other demands.

Regarding the secondary network of the third sector, a *normal* bond was mentioned with a support house, described as a place to carry out activities in several areas. In this network, bonds with housemates were mentioned, as well as an *interrupted* bond with an NGO, considered an important source of support at the beginning of the diagnosis.

Universities, which made up the formal secondary network, were mentioned in the construction of important friendship bonds, as well as bonds related to education and scholarships. Moreover, it is the place for testing and confirming a positive result for HIV.

Market networks were included as they make up the family’s source of income, represented by the employment relationship of people living with HIV and AIDS and also of their families. However, in some individual networks, various types of bonds were built with co-workers. *Interrupted* ­employment ­relationships after the diagnosis of HIV, due to abandonment, mentioning work with sharp material, and also due to dismissal, after being aware of the diagnosis of Tuberculosis, were highlighted.

The construction of the social networks of people living with HIV and AIDS also allows visualizing the sources of support after the diagnosis. In this context, the consolidated networks map shows that the members who offered support were mother, father, spouse/partner, children, brother/sister, other relatives (nephews/nieces and son-in-law), colleagues, and friends. The inclusion of support from the infectious disease professional is highlighted, referred to in two individual networks.

## DISCUSSION

The indicators of the amplitude and density of the primary networks of people living with HIV and AIDS reveal that, in these networks, despite having a median composition, the ties established between the members do not indicate ­intensity in the relationships. However, it should be highlighted that the bonds between the members of the primary network, which characterize density, were not related to aspects of the HIV ­diagnosis, being referred to as bonds of family and social coexistence.

The dynamics of predominantly familiar relationships marks the structure of the networks of people living with HIV and AIDS, which is surrounded by ties of varied nature, allowing us to affirm that it is in the family that the person’s first relational experience is constituted, being the first and most important knot of the networks.

Family ties are not chosen and, even in the rupture or ­interruption of a tie, the family will continue to be a point of reference that always reappears for better or for worse, re-presenting itself as a resource or an obstacle. This web of ­different types of bonds denotes this condition of the family network. Kinship ties also take an important place in the network, characterized by more distant but significant relationships^(8)^.

Corroborating the research findings, the family is always identified as the main component of the social network of ­people living with HIV and AIDS and the main source of daily support for coping with the disease^(2,11–12,19)^, because it constitutes the first space of social coexistence in which an individual is inserted, acting in a decisive way in the other relationships^(20)^.

The presence of strong bonds of friendship in the primary network of people living with HIV and AIDS is highlighted. These friendship relationships are built by the criterion of ­preference, based on affective closeness and, when triggered, are important to meet the needs^(8)^. Studies show that friends are an important source of support for coping with this disease^(11–12,19,21)^.

The role of neighbors in social networks is specified, as they are responsible for physical proximity, and can be reached more quickly in case of necessity^(8)^. In addition to the ­physical ­proximity provided in the neighborhood relationship, the author mentions the importance of this proximity with colleagues, especially those at work. Considering these aspects, studies reveal that, although not being the main source of support, bosses, co-workers and neighbors^(13,21)^ also participate in the social support network for people living with HIV and AIDS.

However, after HIV infection, there are changes in ­friendship cycles, marked mainly by the inclusion of people who are also living with HIV^(22)^, a situation that is similarly experienced by the participants of this study.

There were changes in the dynamics of social relationships in the primary network that are influenced by the HIV diagnosis. Despite extensive networks consisting of family members, ­relatives, friends, and colleagues, few members are aware of the diagnosis. This situation affects these relationships, often not being considered as trusting, creating limits in coexistence and maintenance of confidentiality about the disease.

Primary networks produce imperceptible movements, ­determined by different relational experiences of their ­members, which take place according to everyday requirements. Nevertheless, some positive or negative situations, called critical events, can influence the movement of the entire network, such as a disease, referred to as a negative critical event, which is responsible for interrupting or recruiting some relationships, strengthening or weakening ties^(8)^.

In the case of people living with HIV and AIDS, more than the disease itself, diagnosis confidentiality for members of the primary network can be marked as the critical negative event of their social network. This event can sometimes lead to distance in relationships due to the maintenance of this secrecy, and it can establish new ties and strengthen old ties, especially with those who are aware of the disease, or in the face of some need.

In this regard, it is important to consider that the main reason for diagnosis confidentiality was the fear of prejudice, a feeling still present in the daily life of people living with HIV and AIDS, as corroborated by the findings of other studies in the area^(2,19)^, which show that omission takes place in different spheres, be it in the family, socially or at work.

The fear of suffering prejudice and discrimination for being HIV positive, either from family members, relatives, friends, or colleagues, in different social contexts, reflects on the ­relationships that constitute the social network of people living with HIV and AIDS, resulting in changes in the structure and dynamics of networks, which can influence these people’s daily lives.

Following HIV diagnosis, personal and social responsibilities increase and each person deals with these demands in different ways^(23–24)^. Some may view this situation with ­positive ­dimensions, seeking greater family approximation, practicing self-care, changing habits, while others suffer with daily ­changes, due to fragility in marital relationships, abandonment by the partner, and family distancing. This can result in social ­isolation, as well as the interruption of leisure and work activities, due to the multidimensional social stigma associated with the infection^(3,23–24)^.

Regarding the relationships in the secondary network, more specifically the specialized service, it is possible to ­evidence strengthened bonds with medical professionals, especially the infectious disease specialist, to the detriment of other­ ­professionals. However, consultations were mentioned in various medical specialties (psychiatry, gynecology), in addition to nursing, psychology and dentistry, configuring a multidisciplinary approach.

In the same perspective, the study regarding the satisfaction of people living with HIV and AIDS, with the specialized assistance service in a municipality of Paraná, shows evidence of positive indicators regarding the physician-patient bond, presenting good indicators related to multiprofessional care^(25)^.

Although the Specialized Assistance Service (*SAE*) in HIV/AIDS should provide comprehensive and quality service, to meet the care needs of people living with HIV and AIDS, the health care network shall be articulated, stimulating the integrality and decentralization of care actions^(26–27)^. Thus, other services have to participate in the assistance network for this group, including sharing this care with primary care^(27–29)^.

Although primary care services have been well evaluated by people living with HIV and AIDS, the creation of bonds and the use of these services are weakened, especially due to the incipient offer of HIV-related care. Such aspects can be justified by the fear of breach of confidentiality and by having the perception that a single service is capable of meeting their demands^(28,30)^.

The broad formal secondary network described in the findings allows us to reflect that a single service does not have the entire apparatus to fully meet the needs of people living with HIV and AIDS. The context of these people requires expanded perspectives and an articulated organization of the care network, to meet the multidimensional complexity that involves them.

The collaboration of the study participants to consolidate the information may have been limited, since the object studied is still immersed in a historical and social context marked by stigma and prejudice, which may have influenced the interaction and detachment at the time of the interview, and during the design of social networks. There was also a limitation in the data collection process, due to the lack of a stage for ­approval of the final design of the individual social network after the ­consolidation of the interview and graphic design. This step would allow a more reliable validation of the social network constructed.

It is expected that the study will allow discussions between the actors and sectors of the social networks of people living with HIV and AIDS, as it creates conditions that suggest the importance of articulation and integration between members through relationships of care sharing.

## CONCLUSION

The construction of maps allowed knowing the social network of people living with HIV and AIDS. At the same time, it signaled the uniqueness of living with HIV and AIDS, based on the web of affective, social and institutional bonds ­established, evidencing a daily life that influences and is ­influenced by the relational dynamics and context, with bonds that are organized to meet the needs of these individuals.

Structurally, the primary networks of these people are ­constituted by various bonds, determined by affective and social coexistence, and have the family as the center. Ties established in this network are influenced by the secrecy of HIV diagnosis. The fear of suffering prejudice and discrimination for being HIV positive in the family and social context interferes with the constitution of social networks, resulting in variations in their structure and dynamics and with the potential to influence these people’s daily lives.

The secondary network has a varied composition, with ­stronger ties with the specialized outpatient clinic, but including other health, social and informal service institutions. However, the secondary services are the preferred ones, and they take on the role of specific care for the disease.

In the context of care for people living with HIV and AIDS, knowing and recognizing the actors and sectors that make up the structure of social networks, both by the individuals with HIV and AIDS and by the professionals who follow them, constitutes an important resource to know the existing social capital and, thus, be able to use them in the face of either health or social demands.
